# Authors' Reply

**DOI:** 10.18502/jovr.v16i1.8265

**Published:** 2021-01-20

**Authors:** Siamak Moradian, Marzieh Ebrahimi, Azade Kanaani, Amir Faramarzi, Sare Safi

**Affiliations:** ^1^Ophthalmic Epidemiology Research Center, Shahid Beheshti University of Medical Sciences, Tehran, Iran; ^2^Ophthalmic Research Center, Shahid Beheshti University of Medical Sciences, Tehran, Iran; ^3^Department of Stem Cells, Cell Science Research Center, Royan Institute, Tehran, Iran

Dear Editor,

We would like to thank Dr Arjun Srirampur for his interest in our work. Below please notice our replies:

1. All of our diabetic patients were operated upon due to proliferative diabetic retinopathy (PDR) complications such as non-clearing vitreous hemorrhage and traction retinal detachment but there was no case of rhegmatogenous retinal detachment; therefore no tamponade was used during surgery.

2. None of our patients had uncontrolled IOP during the short follow-up period (two weeks) after surgery in both groups. The corneal epithelial defect (CED) was improved in all eyes except three; and in those three eyes, lateral tarsorrhaphy and application of a lubricant ointment resulted in healing of the CED less than one month after surgery.

